# Memory Deficit in Patients With Temporal Lobe Epilepsy: Evidence From Eye Tracking Technology

**DOI:** 10.3389/fnins.2021.716476

**Published:** 2021-09-07

**Authors:** Guangpu Zhu, Jing Wang, Ling Xiao, Ke Yang, Kailing Huang, Beibin Li, Sha Huang, Bingliang Hu, Bo Xiao, Ding Liu, Li Feng, Quan Wang

**Affiliations:** ^1^Key Laboratory of Spectral Imaging Technology, Xi’an Institute of Optics and Precision Mechanics of the Chinese Academy of Sciences, Xi’an, China; ^2^University of Chinese Academy of Sciences, Beijing, China; ^3^Key Laboratory of Biomedical Spectroscopy of Xi’an, Xi’an Institute of Optics and Precision Mechanics of the Chinese Academy of Sciences, Xi’an, China; ^4^Department of Neurology, Xiangya Hospital, Central South University, Changsha, China; ^5^Paul G. Allen School of Computer Science and Engineering, University of Washington, Seattle, WA, United States; ^6^Department of Neurology, The Third Xiangya Hospital, Central South University, Changsha, China

**Keywords:** eye tracking, memory impairment, temporal lobe epilepsy, electroencephalography, visual attention

## Abstract

**Objective:** To explore quantitative measurements of the visual attention and neuroelectrophysiological relevance of memory deficits in temporal lobe epilepsy (TLE) by eye tracking and electroencephalography (EEG).

**Methods:** Thirty-four TLE patients and twenty-eight healthy controls were invited to complete neurobehavioral assessments, cognitive oculomotor tasks, and 24-h video EEG (VEEG) recordings using an automated computer-based memory assessment platform with an eye tracker. Visit counts, visit time, and time of first fixation on areas of interest (AOIs) were recorded and analyzed in combination with interictal epileptic discharge (IED) characteristics from the bilateral temporal lobes.

**Results:** The TLE patients had significantly worse Wechsler Digit Span scores [*F*(1, 58) = 7.49, *p* = 0.008]. In the Short-Term Memory Game with eye tracking, TLE patients took a longer time to find the memorized items [*F*(1, 57) = 17.30, *p* < 0.001]. They had longer first fixation [*F*(1, 57) = 4.06, *p* = 0.049] and more visit counts [*F*(1, 57) = 7.58, *p* = 0.008] on the target during the recall. Furthermore, the performance of the patients in the Digit Span task was negatively correlated with the total number of IEDs [*r*(28) = −0.463, *p* = 0.013] and the number of spikes per sleep cycle [*r*(28) = −0.420, *p* = 0.026].

**Conclusion:** Eye tracking appears to be a quantitative, objective measure of memory evaluation, demonstrating memory retrieval deficits but preserved visual attention in TLE patients. Nocturnal temporal lobe IEDs are closely associated with memory performance, which might be the electrophysiological mechanism for memory impairment in TLE.

## Introduction

Temporal lobe epilepsy (TLE) is the most common focal epilepsy in adults and is associated with a generalized pattern of cognitive impairment (Elger et al., 2004; Sen et al., 2018). Seventy percent of TLE patients have memory dysfunction, which has been documented to be related to hippocampal sclerosis (Ozkara et al., 2004; Helmstaedter and Kockelmann, 2006; Postma et al., 2020), seizure frequency and duration (Aldenkamp and Arends, 2004; Elger et al., 2004; Black et al., 2010), interictal epileptic activity (Dinkelacker et al., 2016; Ung et al., 2017) and antiepileptic medication (Aldenkamp et al., 2003; Bernardi and Barros, 2004). However, this traditional epilepsy-centric view of cognitive deficits has recently been challenged by observations that cognitive deficits, including memory impairment, may well precede the onset of epilepsy and be viewed as a biomarker for disease development and prognosis assessment (Hermann et al., 2012; Witt and Helmstaedter, 2012, 2015). Thus, sensitive and sensible neurocognitive assessments for longitudinal follow-up in epilepsy are needed to facilitate earlier diagnosis and intervention of TLE.

Conventional memory assessment applied in epilepsy includes the Wechsler Memory Scale (WMS) (Sherman et al., 2012), Hopkins Verbal Learning Test (McAuley et al., 2015), and Rey Auditory Verbal Learning Test (Sveikata et al., 2019). However, high subjectivity, relative inaccuracy, low sensitivity, and poor repeatability limit the clinical application of these assessments. Additionally, increasing evidence suggests that important memory–viewing attention interactions have mostly been ignored in previous research (Voss et al., 2017). Working memory can be used to guide attention and visual search, and attention can in turn determine what information will be fed and encoded into memory (Bahmani et al., 2019). However, the majority of present memory-related scales cannot separate the tightly intertwined effect between visual attention and memory formation (Molitor et al., 2014; Wirth et al., 2017). Observation behavior and associative processing are thus often confused, rendering the interpretation of cognitive deficits ambiguous (Voss et al., 2017).

In contrast, eye tracking technology has become a more widely available and potential technology that can facilitate the traditional memory assessment of cognitive screening in neurological diseases thanks to its millisecond-level sampling rate, quantified measurements, and reliability during the monitoring process (Tao et al., 2020). Moreover, this technology can capture people’s ocular motor information (Pihl-Jensen et al., 2017), reflect complex cognitive processes during tasks (Heuer et al., 2013), help eliminate possible attentional confounding in visual behavior (Chau et al., 2015), and assist in clarifying the underlying mechanism of cognitive impairment disease (Nagasawa et al., 2011; Bastin et al., 2012). Eye tracking technology is confirmed to contribute to the early diagnosis of neurodegenerative diseases. Tadokoro et al. (2021) successfully discriminated cognitive functions among normal control (NC), mild cognitive impairment (MCI), and Alzheimer’s disease (AD) subjects using eye tracking technology. Tabashum et al. (2021) illustrated that pupil size and fixation duration of eye tracking data can be applied for early detection of Parkinson’s Disease (PD). Eye tracking technology and epilepsy also linked closely. Asato et al. (2011) performed visually or memory-guided saccade and anti-saccade tasks in pediatric epilepsy and found that deficits in oculomotor function might contribute to worse complex neuropsychological performance in pediatric epilepsy patients. In children and adults with focal epilepsy, Nagasawa and Lunn confirmed that patients with a long course of epilepsy had higher anti-saccade peak velocity and gain (Nagasawa et al., 2011). A correlation between emotional preference and memory activity in epileptic patients has been identified by a combination of the emotional face recognition task and eye tracking monitoring (Okruszek et al., 2017). Although the door is open, there is still a long way for eye tracking to be developed as a cognition evaluation tool and to find a marker for disease progression and prognosis in epilepsy.

It is worth noting that hippocampal electrical oscillations have been associated with processes of memory consolidation during the sleep stage and might support memory reactivation and redistribution (Rasch and Born, 2013). The dynamic functional alterations of the brain network, such as interictal epileptic discharges (IEDs) in the temporal lobe, might influence seizure-related memory impairment in TLE (Halasz et al., 2019). IEDs are defined as spikes or spike-wave complexes that occur individually or continuously without evidence of seizures (Guo et al., 2018). It is well known that the characteristics of IEDs, including the spike frequency (Aldenkamp and Arends, 2004; Ebus et al., 2012), localization (Wolff et al., 2005; Ebus et al., 2012; Glennon et al., 2016), appearance time (Fonseca et al., 2007; Filippini et al., 2013), and duration of serial spike-wave complexes (Aldenkamp et al., 2005), may have effects on cognition. Higher interictal activity on electroencephalograph (EEG) is negatively correlated with verbal memory performance in mesial temporal sclerosis (Mantoan et al., 2009). With depth electrode monitoring, the effects of hippocampal IEDs on single-neuron activity and declared memory strength have been confirmed, revealing that IEDs might disrupt medial temporal lobe-dependent declarative memory retrieval processes (Reed et al., 2020).

In the present study, a computer-based platform for automated memory assessment with the eye tracker was created. In combination with conventional memory assessment scales, we aimed to compare memory performance between patients and healthy controls. With our Short-Term Memory Game with eye tracking, we separated the attention component of the visual memory tasks and addressed the memory deficits in TLE patients. Additionally, by performing IED analysis during the sleep stage, we tried to provide more electrophysiological evidence for memory impairment in TLE patients.

## Materials and Methods

### Participants

Thirty-four patients (19 females, age 30.65 ± 8.94) with TLE and twenty-eight age-matched healthy controls (16 females, age 32.93 ± 10.01) were included in this study. All participants had normal or corrected-to-normal vision and normal hearing. This study received ethical approval from the Ethics Committee of Xiangya Hospital of Central South University, and all subjects provided written informed consent to participate in the study.

Epilepsy patients were recruited from Xiangya Hospital of Central South University of China with a diagnosis of TLE from October 2017 to March 2018. The diagnosis and classification of epilepsy type were performed by two trained epileptologists and secondarily confirmed by study authors, according to International League Against Epilepsy (ILAE) classification (Berg et al., 2010). Study inclusion criteria included (1) patient participants who met the criteria for the classification of TLE epilepsy and (2) full-scale IQ ≥ 70. All patients were tested while taking routine medications. Three of them did not start medications when included in the study. Twenty of them received monotherapy, among which seven patients were taking oxcarbazepine, six used valproic acid, five used carbamazepine, one used lamotrigine and one used topiramate. The rest eleven patients received polytherapy, they used two or three drugs of oxcarbazepine, valproate acid, levetiracetam, lamotrigine, carbamazepine and other antiseizure drugs. The exclusion criteria were as follows: (1) the patient’s age was less than 18 or more than 70 years; (2) the patient had other neurological or psychotic disorders or comorbidities; and (3) the imaging MRI was negative, but hippocampal sclerosis was included.

Twenty-eight healthy individuals matched in age, sex, and level of education to the patients with TLE were also recruited from the community. Healthy individuals with neurological or psychiatric diseases, and drug addiction were excluded.

### Wechsler Memory Assessment

All tests and questionnaires were administered by the same neuropsychologist expert (MGV) blinded to the clinical information. For the traditional memory assessment, we applied two tests of Wechsler Memory Scales-Chinese Revision (WMS-RC) (Wechsler, 1945; Gong et al., 1989). (1) Digit Span: This is a verbal memory test. The participant is instructed to recall the sequence of numerical digits to the experimenter after hearing them. The sequence becomes increasingly longer in each trial. The test ends when the participant cannot accurately recall the full sequence of digits. The participant’s score is the longest number of sequential digits that can accurately be remembered. The Digit Span test can also be given backward, meaning that the participant is asked to recall the sequence in reverse order. (2) Visual Recognition: This is a non-verbal, visual short-term memory test. Eight cards are shown to the participant for 30 sec, including graphics, Chinese characters and symbols. Then the participant is asked to recognize the previous eight cards in 28 cards. The more cards the participants recognize, the higher the score. Both tests examine memory of the participants, but the Digit Span test is related to verbal memory, while the Visual Recognition test involves visual memory.

### Cognitive Oculomotor Tasks

#### Game Design

To increase the robustness and reproducibility of memory assessment, we created an automated computer-based memory assessment platform that provides game-like interaction. This platform was applied simultaneously with a visual attention tracking system, the Tobii Glass II wearable eye tracker.

This automated platform was adapted from Li et al. (2018), implemented with Unity and C#, and deployed on Windows Laptop computers. We had three tasks in total, including Shifting Game for testing rule learning and adaption, Inhibitory Control Game for testing executive function, and Short-Term Memory Game. In this paper, we focused on the memory game, and the other two will be analyzed and published in separate papers. To test the memory for different types of stimuli, we used human front-facing images and fractals as memory targets and distractors. Forty human front-facing images were downloaded from the Chinese University of Hong Kong (CUHK) student database^1^ and 40 fractals were downloaded online with control of image resolution, illumination, and complexity. We evaluated our design by conducting a pilo*t*-test with six adults with no vision or cognitive disabilities. The results indicated that the image stimuli were compatible in terms of attractiveness. The design of social and non-social tasks was analogous.

**FIGURE 1:**
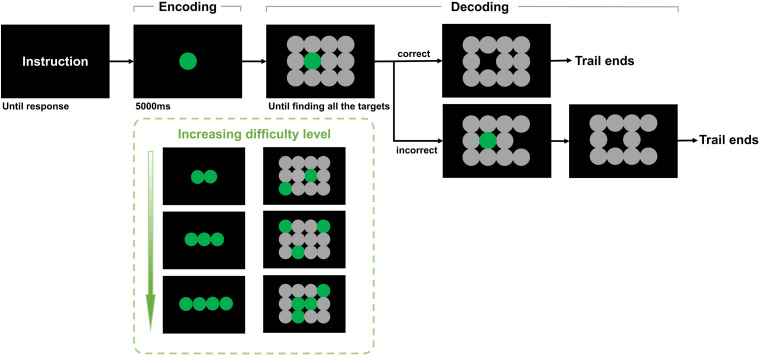
Short-term memory game with eye tracking. There are four trials in the task. The first trial consists of three parts. First, the instruction is displayed on the screen to explain the rules. Then, one target is displayed, and the participant needs to remember the target within 5,000 ms (encoding). Next, the participant needs to recall and click on the target from 4 × 3 objects (decoding). If the selection is correct, the selected object disappears, and the participant enters the next trial. If not, the selected image disappears, and the participant can enter the next trial until the target is clicked. The second, third, and fourth trials presented 2, 3, and 4 targets, respectively, that needed to be remembered and identified by the participant.

**FIGURE 2:**
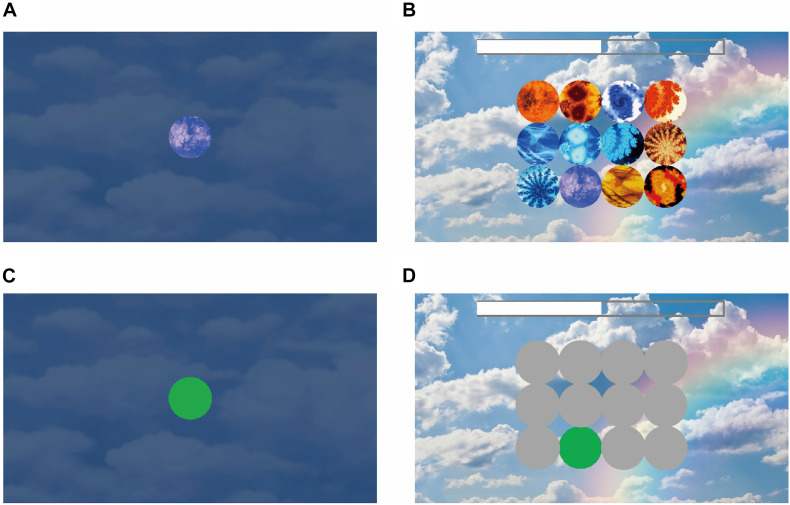
Experiment stimuli in the short-term memory game. **(A)** Encoding phase. In this example, the participant has only one target to remember. This target shows on the screen for 5 s. **(B)** Decoding phase. There were 4 × 3 objects on the screen, and the participant needed to click on the target that they had seen during the encoding phase. During the encoding and decoding phase, eye movement was recorded with an Eye Tracker. **(C)** AOI of the target during the encoding phase. **(D)** AOIs of the target (in green) for recognition and distractors (in gray).

In the encoding phase of the memory game, one to four memory targets are presented for 5 s along the middle horizontal line of the iPad screen for participants to memorize. Then, in the decoding phase, the memory targets disappear, and 12 potential answers are presented on the screen in a 4 × 3 array. The participants must select the target(s) from the previous screen to proceed to the next, more difficult level (Figures 1, 2). Human voices are provided as feedback for clicking the correct answer “Yes” or incorrect answer “No” with no latency. Game completion time was recorded and analyzed.

#### Eye Tracking

All participants underwent the Wechsler memory assessment and the Short-Term Memory Game. The hardware system was Tobii Glass II. We had a 5-point on-screen calibration system and confirmed that all the participants had < 100-pixel errors on the computer screen (which was smaller than the size of the target size on our game memory system). Data analysis was applied with Tobii Pro Lab (version 1.123). We defined the area of interest (AOI) as each target in the memory encoding phase and each object in the memory decoding phase (Figures 2C,D). We screenshotted each layout of the computer screen and aligned it with each participant’s scene camera video to determine the gaze point position on the computer screen. Then, we determined how the gaze position landed into the AOIs. After prepossessing with the AOIs, we exported the visit counts, total visit time, average visit time, and time of first fixation as the output variables. A visit was defined as eye gaze entering one AOI and the successive fixation(s) within the AOI. The eye gaze moving out of the AOI referred to the end of the visit. Total visit time was the sum of looking duration of all visits in each AOI. The visit counts were how many times eye gaze entering and moving out of each AOI. Average visit time was defined as the total visit time divided by the visit counts. And time of first fixation was defined as the latency of the first visit to the AOI, counting from the start time of each screen display. We processed the eye tracking data with MATLAB (Version 2015b).

### EEG Recordings

Video EEG (VEEG) monitoring is a procedure usually done during an inpatient hospital stay using continuous recording of EEG and video. The goal is to record patients’ daily events and determine if they are epileptic seizures or some other condition. For epileptic seizures, the VEEG can help determine the frequency and the type of seizure and may pinpoint the area in the brain where they begin. All patients underwent a 580-G2CGSS VEEG monitoring system (Biologic of America), but healthy controls did not. This system has a sampling rate of 500 Hz and a bandwidth of 0.01. The room had a digital camera mounted near the ceiling and focused on the patients in the bed. The 20 Ag/AgCl electrodes (10/20 system) were attached to the scalp with conductive cream. The electrode wires were connected to a 4-by-7-inch transmitter. Attached to the transmitter was a cord connected to the computerized recording system. The monitoring time was 24 h, including awake and sleep stages (at least one sleep cycle was included). We asked the patient or the person with the patient to press the epileptic alarm button when the suspected epileptic seizure occurred.

The record was reviewed by two experienced neurophysiology consultants independently. IEDs are defined as described by ILAE (Nicolai et al., 2012). The full report of interictal EEG features during non-rapid eye movement sleep (NREM) with a 12-h span (from 8 p.m. on the first day to 8 a.m. on the second day) was evaluated. The total number of IEDs from the bilateral temporal lobes during NREM over a 12-h span was calculated.

### Statistical Analyses

Participant’s characteristic information were compared using *χ*^2^ analyses for categorical variables and independent-sample *t*-tests for continuous variables between patients with TLE and healthy controls. Scores of Wechsler memory tests obeyed a normal distribution and were analyzed with the Linear Mixed Model between patients and healthy groups. Eye tracking metrics obeyed a normal distribution and were compared using the Linear Mixed Model between two groups. Group (TLE patients/healthy controls) was included as a fixed-effect factor, and age was included as a covariate, whereas individual identifier was included as a random-effect factor. Associations between variables of interest were examined using the Pearson’s r correlation coefficient analysis. A two-tailed *P*-value was calculated for all tests and *P*-values of less than 0.05 were considered as statistically significant. Statistical analysis was performed using the SPSS software package (ver. 24.0; SPSS Inc., Chicago, Illinois, United States). Statistical analysis was performed using the SPSS software package (ver. 24.0; SPSS Inc., Chicago, Illinois, United States).

## Results

### Demographics

All participants were paired separately by age and gender with no differences in these categories. There were also no differences in the level of education between the patient and control groups. Other characteristics, including neuropsychological tests, are summarized in Table 1.

**TABLE 1:** Characteristics of participants.

	Controls (*n* = 28)	Patients (*n* = 34)	Cohen’s d
N	28 (16)	34 (19)	
Age	32.92 ± 10.01	30.65 ± 8.94	0.24
Education (years)	3.11 ± 1.23	2.97 ± 1.17	0.12
Age at onset of epilepsy	−	20.32 ± 12.36	−
Duration of epilepsy (years)	−	10.32 ± 9.51	−
Seizure frequency (every year)	−	30.53 ± 86.81	−
*AEDs*			
No AEDs	−	3	−
Single AEDs	−	20	−
Combination of AEDs used	−	11	−
Digit Span	12.48 ± 4.32	9.82 ± 4.12	0.63
Visual recognition	10.32 ± 3.07	8.91 ± 3.34	0.44

*AED, antiepileptic drug.*

### Wechsler Memory Assessment

**FIGURE 3:**
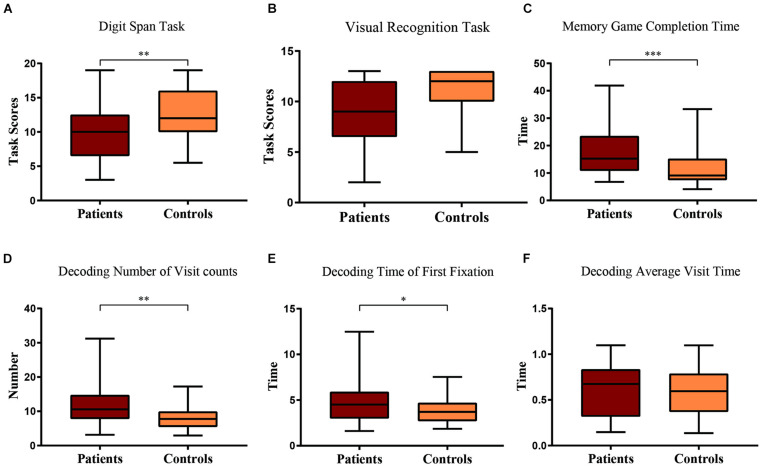
Boxplot of patient and control scores in **(A)** Digit Span task and **(B)** Visual Recognition task. Their comparison of **(C)** game completion time, **(D)** number of visits, **(E)** time of first fixation, and **(F)** average visit time in the decoding phase of the short-term memory game. Statistical significance for each task between groups is indicated by asterisk(s) (^∗^*p* < 0.05, ^∗∗^*p* < 0.01, ^∗∗∗^*p* < 0.001).

In the Digit Span Backward task of the WMS, with a Linear Mixed Model with clinical diagnosis as the factor and age as the covariate, we found that the number of items memorized by patients was significantly less than that of the control group [*F*(1, 58) = 7.49, *p* = 0.008], and age was significant [*F*(58) = 4.44, *p* = 0.040; Figure 3A].

Similarly, in the visual recognition task of the WMS, with a Linear Mixed Model with clinical diagnosis as the factor and age as the covariate, we found that the patients had a marginally worse performance than the control group [*F*(1, 58) = 3.77, *p* = 0.057], and age was also marginally significant [*F*(58) = 3.75, *p* = 0.058; Figure 3B].

### Short-Term Memory Game and Eye Tracking

Through automated computer-based assessment with eye tracking, during the process of remembering the visual stimuli, the Linear Mixed Model showed that the patients and healthy participants spent the same amount of time looking at the target items [*F*(1, 56) = 0.049, *p* = 0.826; Table 2]. Given that there was no difference between the patients and healthy participants during the remembering phase, we only included the eye tracking data during the recall phase of the experiment. While trying to recall and find the memorized items, the Linear Mixed Model showed that the patients took significantly longer time to find the target items [*F*(1, 57) = 17.30, *p* < 0.001], and age as the covariate was significant [*F*(1, 57) = 32.19, *p* < 0.001; Table 2 and Figure 3C].

**TABLE 2:** Differences in eye tracking indicators between TLE patients and healthy controls and VEEG data.

		Controls (*n* = 28)	Patients (*n* = 34)	Cohen’s d
ET	*Encoding*			
	Total visit time	2.88 ± 1.35	2.79 ± 1.25	0.07
	*Decoding*			
	Memory game completion time	12.29 ± 7.35	17.86 ± 9.36	−0.65
	Number of visit counts	8.98 ± 5.78	11.73 ± 6.44	−0.45
	Time of first fixation	4.17 ± 2.53	5.89 ± 4.35	−0.47
	Average visit time	0.61 ± 0.26	0.90 ± 1.71	−0.23
VEEG	The total number of spikes	−	24.90 ± 41.35	−
	The number of spikes per sleep cycle	−	2.76 ± 5.53	−

*TLE, temporal lobe epilepsy; ET, eye tracking; VEEG, video electroencephalography.*

During recall, eye tracking data showed that patients had more visit counts [*F*(1, 57) = 7.58, *p* = 0.008], the first fixation on target was longer [*F*(1, 57) = 4.06, *p* = 0.049] during the process of trying to find the memorized images, and age was a significant covariate (*p* < 0.05; Table 2 and Figures 3D,E). However, the average observation time per visit was not different between groups [*F*(1, 57) = 0.094, *p* = 0.760], and the difference was not significant (*p* > 0.05; Table 2 and Figure 3F).

**FIGURE 4:**
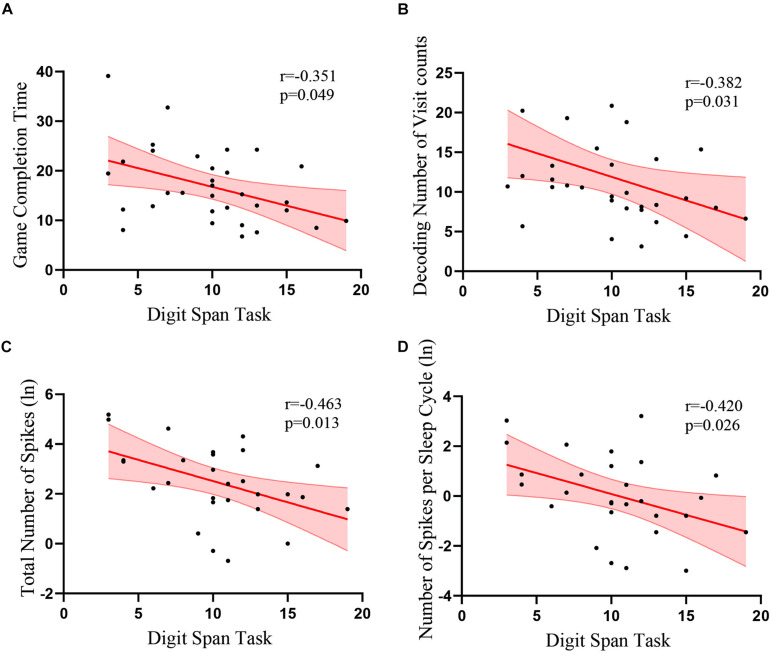
Correlations between the scores of the Wechsler Digit Span task and eye tracking data and EEG recordings. Correlations between the scores of Digit Span task and **(A)** game completion time **(B)** numbers of visits during the memory decoding phase **(C)** total number of spikes **(D)** number of spikes per number. Red lines indicate linear fits. Light red regions indicate standard error of the means.

In the control group, there was no correlation between the assessment and the time to find the target items or any eye tracking measures in the memory game, including number of visits, total visit time, average visit time, and time of first fixation (*p*s > 0.1). In the patient group, there was no correlation between the Wechsler Visual Recognition scores and the time to find the target items or all eye tracking measures during the recall phase (*p*s > 0.1). However, in the patient group, there was a significant correlation between the scores of the Wechsler Digit Span task and memory game completion time [*r*(32) = −0.351, *p* = 0.049; Figure 4A]. The visit count in eye tracking [*r*(32) = −0.382, *p* = 0.031] was also negatively correlated with Digit Span task scores (Figure 4B). Time of first fixation [*r*(32) = −0.342, *p* = 0.055] was marginally correlated with Digit Span task scores, but average visit time [*r*(32) = −0.087, *p* = 0.636] was not correlated.

### Relationship of VEEG and Memory Deficits

We analyzed the correlation between the IEDs during sleep and the cognitive performance of the patients with TLE. We found that the performance of the patients in the Digit Span task was negatively correlated with the total number of spikes in the temporal lobe [*r*(28) = −0.463, *p* = 0.013; Table 2 and Figure 4C] and the number of spikes per sleep cycle [*r*(28) = −0.420, *p* = 0.026; Table 2 and Figure 4D]. However, there was no correlation between the VEEG data and the eye tracking data (*p* > 0.3).

## Discussion

In this study, we investigated short-term memory deficits among adult patients with TLE and found that TLE patients had worse Digit Span scores on the WMS test. Second, monitoring the Short-Term Memory Game through an automated and quantitative eye tracking system further confirmed similar memory impairment in TLE patients. Moreover, the eye movement data showed that there was no difference in looking time during the encoding phase between the patients and the control group, which indicated that visual attention was not the main factor influencing the memory encoding process in TLE patients. On the other hand, our EEG data showed that the number of accumulative abnormal IED spikes from the temporal lobe during sleep was negatively correlated with patients’ memory performance, which strengthened the argument that IEDs may contribute to memory impairment in epilepsy.

With two tasks of WMS, we found that TLE patients had significantly worse Digit Span scores (*p* = 0.008), but their visual recognition task scores were only marginally lower than those of the control group (*p* = 0.057). The difference between these two Wechsler short-term memory tasks was that visual attention was heavily involved during the visual recognition task but not in the Digit Span task. Visual attention is a pervasive behavior that fundamentally determines the information available for memory, but it is usually not measured in the majority of memory experiments (Hollingworth et al., 2001; Zelinsky and Loschky, 2005). In most circumstances, the attention module during memory tasks cannot be accurately evaluated due to lack of control for viewing behavior assessment. Eye tracking in memory paradigms was helpful in resolving this issue and separating the effect of visual attention (Douglas et al., 2017; Wirth et al., 2017). In our eye tracking experiment, we designed a visual memory game and tracked the eye movement of all participants during the whole memory game. We found that in the memory encoding process, there was no significant difference between TLE patients and healthy controls in total visit time and total visit counts, which means that both groups performed actively in visual attention and were target focusing. However, in the following decoding period, the patients took significantly longer to find the memorized items and had more visit counts to retrieve them, showing that the TLE patients were not able to retrieve or identify the target item as efficiently as the control group. We did not find any correlation between the Wechsler visual recognition scores and the time to find the target items or any eye tracking measures in the memory game, suggesting that visual attention might be unaffected in memory encoding in TLE patients, and this finding was in line with the finding that the TLE group did not perform significantly worse than the control group on the Wechsler visual recognition test.

Nevertheless, including the total looking duration, time of first fixation and visit counts in the analysis, we found a significantly longer memory game completion time and more visit counts in patients with TLE than in controls. Further analysis showed that the longer game completion time in the eye-tracking memory game of TLE was due to more visit counts but not the average looking time per visit. This means that TLE patients can notice the target item as well as the normal controls when they performed the recall task. However, they were not able to pick them out from the other distracting objects. Our results implied that the deficit might be due to memory retrieval or identification problems but not a lack of attention. Moreover, differences were significant in memory game completion time and visit counts between TLE patients and controls, and there was a significant correlation between the scores of the Wechsler Digit Span task and memory game completion time as well as the visit count in TLE patients. These results suggested that automated memory assessment platform with eye tracking has the potential to be a valid and efficient method to complement assessing attention and memory performance in TLE patients.

Increasing evidence has suggested that IEDs in the temporal lobe might contribute to memory deficits in TLE patients. Hoameng found that in patients with left medication refractory epilepsy, spikes outside the seizure onset zone impacted the memory encoding process and reduced the odds of word retrieval (Ung et al., 2017). Jonathan confirmed that hippocampal IED in humans may disrupt memory maintenance and retrieval but not affect encoding (Kleen et al., 2013). In addition, the appearance time of spike onset may be correlated with different extents and types of cognitive impairment. Studies have shown that a high nocturnal spike index is associated with poor cognitive development (Baglietto et al., 2001; Nicolai et al., 2007). Children with electrical status epilepticus during sleep (ESES) are at the severe end of the spectrum of neuropsychiatric abnormalities associated with long-term IEDs in sleep. In our study, we investigated the effect of spikes on memory function and found that nocturnal IEDs of TLE appeared to be significantly negatively correlated with Digit Span backward test scores, which was in line with a previous study showing that nocturnal IEDs were associated with transient memory impairments (Baglietto et al., 2001; Nicolai et al., 2007). However, this association did not occur between sleep spikes and eye tracking data, suggesting that nocturnal temporal lobe IED may disrupt memory maintenance in TLE patients but has no influence on visual guided attention.

The present work is limited in several ways but lays the groundwork for additional important future studies. Although manual spike identification is a valid method for IED analysis in the field, we believe that a more effective and accurate automated algorithm for spike detection is still needed. The effect of anti-epilepsy medication on cognition is also a potential confounding factor but was not assessed in our study design, which may limit the statistical power. Considering relatively small samples and epilepsy variables, further studies with more patients undergoing continuous electroencephalography and eye tracking monitoring during cognitive tasks are warranted.

## Conclusion

In summary, the correlation between eye tracking data and the score of traditional WMS in TLE patients identified that the eye tracking-based automated memory assessment platform can be an assistive tool to assess visual attention and memory performance in TLE. Our study also provided evidence that TLE patients had retrieval deficits in the Short-Term Memory Game, but visual attention was preserved. Nocturnal spike analysis demonstrated a significantly negative correlation with short-term memory, supporting the idea that IEDs may disrupt cognitive processes related to the underlying tissue and its function in epilepsy. Notwithstanding the limitations of this study, our results offer a starting point that eye tracking-based cognitive tests have potential as a screening tool for preclinical cognitive measurement and long-term monitoring with minimal combined variables.

## Data Availability Statement

The raw data supporting the conclusions of this article will be made available by the authors, without undue reservation.

## Ethics Statement

The studies involving human participants were reviewed and approved by the Ethics Committee of Xiangya Hospital of Central South University. The patients/participants provided their written informed consent to participate in this study.

## Author Contributions

GZ contributed to drafting of the manuscript for content and interpretation of data. JW, KY, SH, and DL contributed to the acquisition of data. LX contributed to data analysis. KH and BH contributed to revision of the manuscript. BL and BX contributed to the conceptualization and study design. LF contributed to study design and revision of the manuscript. QW contributed to study design and data analysis. All authors contributed to the article and approved the submitted version.

## Conflict of Interest

The authors declare that the research was conducted in the absence of any commercial or financial relationships that could be construed as a potential conflict of interest.

## Publisher’s Note

All claims expressed in this article are solely those of the authors and do not necessarily represent those of their affiliated organizations, or those of the publisher, the editors and the reviewers. Any product that may be evaluated in this article, or claim that may be made by its manufacturer, is not guaranteed or endorsed by the publisher.

## Footnotes

1
http://mmlab.ie.cuhk.edu.hk/archive/facesketch.html
